# Transcriptome Profiling Reveals Important Transcription Factors and Biological Processes in Skin Regeneration Mediated by Mechanical Stretch

**DOI:** 10.3389/fgene.2021.757350

**Published:** 2021-09-29

**Authors:** Wei Liu, Shaoheng Xiong, Yu Zhang, Jing Du, Chen Dong, Zhou Yu, Xianjie Ma

**Affiliations:** Department of Plastic and Reconstructive Surgery, Xijing Hospital, Fourth Military Medical University, Xi’an, China

**Keywords:** transcriptome, mechanical stretch, hub genes, tissue expansion, skin regeneration, transcription facotrs

## Abstract

**Background:** Mechanical stretch is utilized to promote skin regeneration during tissue expansion for reconstructive surgery. Although mechanical stretch induces characteristic morphological changes in the skin, the biological processes and molecular mechanisms involved in mechanically induced skin regeneration are not well elucidated.

**Methods:** A male rat scalp expansion model was established and the important biological processes related to mechanical stretch-induced skin regeneration were identified using Gene Ontology (GO) analysis, Kyoto Encyclopedia of Genes and Genomes (KEGG) analysis, and gene set enrichment analysis (GSEA). Analysis was also conducted by constructing a protein–protein interaction (PPI) network, identifying key modules and hub genes, determining transcription factor (TF)-mRNA regulatory relationships, and confirming the expression pattern of the TFs and hub genes.

**Results:** We identified nine robust hub genes (CXCL1, NEB, ACTN3, MYOZ1, ACTA1, TNNT3, PYGM, AMPD1, and CKM) that may serve as key molecules in skin growth. These genes were determined to be involved in several important biological processes, including keratinocyte differentiation, cytoskeleton reorganization, chemokine signaling pathway, glycogen metabolism, and voltage-gated ion channel activity. The potentially significant pathways, including the glucagon signaling pathway, the Wnt signaling pathway, and cytokine–cytokine receptor interaction, were distinguished. In addition, we identified six TFs (LEF1, TCF7, HMGA1, TFAP2C, FOSL1, and ELF5) and constructed regulatory TF–mRNA interaction networks.

**Conclusion:** This study generated a comprehensive overview of the gene networks underlying mechanically induced skin regeneration. The functions of these key genes and the pathways in which they participate may reveal new aspects of skin regeneration under mechanical strain. Furthermore, the identified TF regulators can be used as potential candidates for clinical therapeutics for skin pretreatment before reconstructive surgery.

## Introduction

The use of tissue expansion in burn scar excision, implant-based breast reconstruction, scalp defects, nasal reconstruction, and treating other deformities has become increasingly widespread in plastic reconstructive surgery (Karimi et al., 2019). Tissue expansion is a physiological process characterized by the ability of skin to increase its superficial area in response to mechanical stretch. The external forces exerted by tissue expanders directly alter the cell shape within multilayered skin. Alterations in cell shape subsequently provide feedback to guide cell behavior and modulate cell fate and position, eventually resulting in histological changes in the expanded skin. Mechanical stretch activates a local molecular change to convert physical cues to biological responses, such as mechanosensitive ion channels, G-protein coupled receptors, protein kinases, integrin–matrix interactions, and other membrane-associated signal transduction molecules (Razzak et al., 2016). Biological signals then affect gene expression by initiating signaling cascades, which ultimately leads to skin regeneration. Several studies have demonstrated the occurrence of molecular alterations and related biological processes during tissue expansion (Aragona et al., 2020; Ledwon et al., 2020). However, the biological processes and molecular mechanisms involved in mechanically induced skin regeneration have not been well elucidated.

Skin regeneration associated with tissue expansion is a biological process accompanied by gene transcriptional changes (Ledwon et al., 2020; Park et al., 2020). Physiologically, transcriptional regulation and molecular signaling involve precise gene activation and suppression (Tajik et al., 2016). Changes in gene expression by mechanical stretch are accurately regulated under physiological conditions. As such, high-throughput sequencing approaches have led to the use of multidimensional insights to elucidate key mediating molecules or significant pathways related to mechanical stretch-induced skin regeneration during tissue expansion. In this study, we established a male rat scalp expansion model and performed transcriptome sequencing for expanded, sham operated, and normal skin samples. Bioinformatics analyses were conducted to identify key differentially expressed genes (DEGs). Furthermore, we conducted functional enrichment analysis using DEGs activated by mechanical stretch. We performed Gene Ontology (GO) analysis, Kyoto Encyclopedia of Genes and Genomes (KEGG) analysis, and gene set enrichment analysis (GSEA) to investigate the possible mechanism by which genes are involved in mechanical stretch-induced skin regeneration. We constructed a protein-protein interaction (PPI) network to reconstruct the proteins encoded by DEGs and predict key modules. Hub genes were identified and TF-mRNA regulatory relationships were reconstructed using DEGs. Thus, we herein used a comprehensive biological pipeline to explore key molecular signatures in newly grown skin during tissue expansion. These discoveries provide novel insights into the molecular mechanisms of skin regeneration because of mechanical stretch.

## Materials and Methods

### Animal Experiments

Six-week-old male Sprague–Dawley rats were purchased from the Experimental Animal Center of the Air Force Medical University (Xi’an, Shaanxi, China) and fed under specific pathogen-free conditions. The experiment was approved by the animal experiment ethics committee of the Air Force Medical University. A round 1 ml silicone expander with a diameter of 1 cm was customized by Wanhe Plastic Materials Co, Ltd (Guangzhou, China). Twelve rats were randomly divided into three groups: the expanded group (n = 4), the sham operated group (*n* = 4), and the normal control group (*n* = 4). The surgery for the expanded group was performed as described in our previous study (Liu et al., 2020). One milliliter of silicone expander was placed under the scalp in the preperiosteal plane. The sham operated group underwent the same surgical process except for the placement of a silicone sheet without saline injection. The normal control group was kept intact. Then, 1 ml sterilized saline was injected to flatten the silicone expander. Sterilized saline was injected every 2 days from the seventh day after surgery in the expanded group (1 ml per injection) until the silicone expander was enlarged to a volume of 9 ml at day 28. The day on which the surgery was performed was defined as day 0.

All analyses were performed on the skin located on the top of the dome induced by the skin expander, since this region experiences the highest strain. The specimens were collected at 48 h after the last injection.

### Histological Evaluation

Scalp tissue samples were fixed in 10% neutral buffered formalin and embedded in paraffin. Then, 3 μm-thick sections were sliced and prepared for hematoxylin and eosin (H and E) staining. Epidermal and dermal thickness was vertically measured every 200 μm. Six images were taken per slide to calculate the mean measurement per slide. The relative epidermal and dermal thicknesses of the scalp samples were measured using ImageJ software (National Institutes of Health, Bethesda, MD, United States).

### Total RNA Extraction, Quality Examination, and Library Preparation

RNA extraction, quality examination and library preparation were performed by Annoroad Gene Technology Co. Ltd. (Beijing, China; http://en.annoroad.com/). Total RNA was extracted using Trizol reagent. RNA purity was checked using the KaiaoK5500® Spectrophotometer (Kaiao, Beijing, China). RNA integrity and concentration were assessed using the RNA Nano 6000 Assay Kit of the Bioanalyzer 2,100 system (Agilent Technologies, Santa Clara, CA, United States). A total of 2 μg RNA per sample was used as input material for RNA sample preparation. Sequencing libraries were generated using NEBNext® Ultra™ RNA Library Prep Kit for Illumina® (#E7530L, New England BioLabs Inc., Ipswich, MA, United States) following the manufacturer’s recommendations, and index codes were added to attribute sequences to each sample.

### mRNA Library Construction and Sequencing

Paired-end RNA sequencing (RNA-seq) analysis was performed by Annoroad Gene Technology Co. Ltd. (http://www.annoroad.com/) using Illumina HiSeq 4,000. Low-quality reads containing adaptors and with high unknown base content (>5%) were removed from the analysis. The RNA-seq reads were mapped using HISAT software (v0.1.6-beta). The sequencing reads were aligned to a reference sequence using Bowtie2 (v2.2.5) and the gene expression level was calculated with the RSEM software package (v1.2.12). mRNAs that were not detected in any sample (read count <1) were excluded from the downstream analysis.

### Library Examination

The RNA concentration of the library was measured using Qubit® RNA Assay Kit in Qubit® 3.0 for preliminary quantification and then diluted to 1 ng/ μL. Insert size was assessed using the Agilent Bioanalyzer 2,100 system (Agilent Technologies), and the qualified insert size was accurately quantified using the Step One Plus™ Real-Time PCR system (valid library concentration >10 nM).

### Library Clustering and Sequencing

Clustering of the index-coded samples was performed on a cBot cluster generation system using HiSeq PE Cluster Kit v4-cBot-HS (Illumina, San Diego, CA, United States) according to the manufacturer’s instructions. One sham operated sample and one normal control sample were deleted due to the clear separation between them and other samples in their own groups. After cluster generation, the libraries were sequenced on an Illumina platform (Annoroad Co. Ltd.) and 150 bp paired-end reads were generated.

### Data Filtering and Alignment

The Perl scripts were used to remove low-quality reads from the original data (raw data). The Perl scripts were submitted on GitHub at https://github.com/mdshw5/fastqp. The reference genomes and annotation files (Ensembl, v. Rnor 6.0.87) were downloaded from the ENSEMBL database (http://www.ensembl.org/index.html). Bowtie2 v2.2.3 was used to build the genome index, and clean data were then aligned to the reference genome using HISAT2 v2.1.0. The read counts for each gene in each sample were counted by HTSeq v0.6.0, and the fragments per kilobase million mapped reads (FPKM) were then calculated to estimate the gene expression levels.

### Differential Gene Expression Analysis

DEGseq v1.18.0 was used for differential gene expression analysis. DESeq2 estimated the expression level of each gene in each sample by linear regression. The p-value was then calculated via Wald test. Finally, the p-value was corrected by the Benjamini–Hochberg method. Genes with q ≤ 0.05 and |log2_ratio| ≥ 1.2 were classified as DEGs. Volcano plots of DEGs were prepared using the “ggplot2” library in R.

### Principal Component Analysis

PCA is used to reduce the dimensionality of large datasets and increase interpretability while minimizing information loss. PCA of the transcriptome data was performed using the “prcomp” function in “stats” package to assess resemblance between samples. The obtained results were visualized in R using the “scatterplot3d” package.

### GO and KEGG Analysis

Metascape (http://metascape.org) enables the extraction of abundant annotations, as well as the identification of enriched pathways and the construction of PPI networks from lists of genes and protein identifiers (Zhou et al., 2019). In this study, Metascape was used to conduct GO and pathway enrichment analysis of DEGs (p-value < 0.01, minimum count of 3, and enrichment factor >1.5). The GO annotations (version:1.2, http://purl.obolibrary.org/obo/go/go-basic.obo) for the biological process, cellular component, and molecular function categories and KEGG pathways (version: 99.0) (Kanehisa and Goto, 2000) were enriched based on the Metascape online tool. Functional enrichment analysis was also performed by using GO (version:1.2)/KEGG (version:99.0) tools of Hiplot (https://hiplot.com.cn/advance/clusterprofiler-go-kegg), a comprehensive web platform for scientific data visualization. The most statistically significant term within a cluster was considered to represent the cluster.

### GSEA

GSEA (https://software.broadinstitute.org/gsea/index.jsp) was performed using GSEA software version 2.2.2.0, which detects whether a series of a priori defined biological processes were enriched in the gene rank derived from DEGs between the expanded group and the sham-operated group. We used the H, C2, and C5 collections from the Molecular Signatures Database (MSigDB v5.0). A threshold of *p* < 0.05 was applied for the analysis.

### PPI Network Analysis

To further clarify the interactive relationships among DEGs, PPI network analysis was performed using the Search Tool for the Retrieval of Interacting Genes (STRING) database (http://string-db.org/). PPIs derived from experiments and databases with a combined score >0.4 were reserved for further analysis. Cytoscape software version 3.8.0 was used to display the selected PPI networks (http://www.cytoscape.org/). To infer more biologically interpretable results, the Molecular Complex Detection (MCODE) tool was used to search for high modularity clusters within the network (degree cutoff = 2, node score cutoff = 0.2, k-score = 2, maximum depth = 100). Clusters that consisted of at least 10 nodes and had an MCODE score of at least five were considered significant. CytoHubba was utilized to identify the top hub genes, which were further selected and ranked by integrating 12 topological methods. All nodes not connected to the main network were excluded from this analysis to obtain reliable results. To identify pathways associated with DEGs in each cluster, we used the ClueGO plugin of Cytoscape.

### TF Identification and TF–mRNA Regulation Relationship Construction

The Animal Transcription Factors Database (TFDB) provides comprehensive annotation and classification of TFs and cofactors; as such, TFDB was employed to identify interactive transcription factors (Hu et al., 2019). Then, ChIP Enrichment Analysis (ChEA): Transcription Factor Binding Site Profiles (Lachmann et al., 2010), JASPAR Predicted Transcription Factor Targets (Fornes et al., 2020), and TRANSFAC Curated Transcription Factor Targets (Matys et al., 2003) were used to predict target genes. The predicted target genes were compared with DEGs to obtain the overlapping DEGs, and the TF–mRNA interactions were visualized by Cytoscape.

### Quantitative Polymerase Chain Reaction Analysis

To reveal the specific hub genes and TFs involved in skin regeneration, we selected the expanded and sham operated groups for further expression verification. Total RNA was extracted from the expanded and sham operated groups using Trizol reagent (Invitrogen, Camarillo, CA, United States). Briefly, 100 mg scalp skin tissue were homogenized in 1 ml Trizol reagent. Post-centrifugation, RNA was extracted with chloroform and precipitated with isopropyl alcohol. Isolated RNA samples were then reverse transcribed into cDNA using a cDNA synthesis kit (Takara, Shiga, Japan) following standard protocols. Quantitative gene expression was performed using synthetic primers and TB Green (Takara). Hub genes (CXCL1, NEB, ACTN3, MYOZ1, ACTA1, TNNT3, PYGM, AMPD1, and CKM) and TFs (LEF1, TCF7, HMGA1, TFAP2C, FOSL1, and ELF5) were included. The primer sequences are listed in Supplementary Table S1. GAPDH was used as an internal reference when analyzing gene expression.

### Statistical Analysis

The differences in expression between the expanded and sham group tissues were calculated using *t*-test or the Mann–Whitney U test. The results are presented as the mean ± standard deviation (SD) from at least three experimental repeats. The data were plotted using GraphPad Prism 7.0 (GraphPad Software, San Diego, CA, United States).

## Results

### Mechanical Stress Induced Histological Alteration of Expanded Skin

**FIGURE 1:**
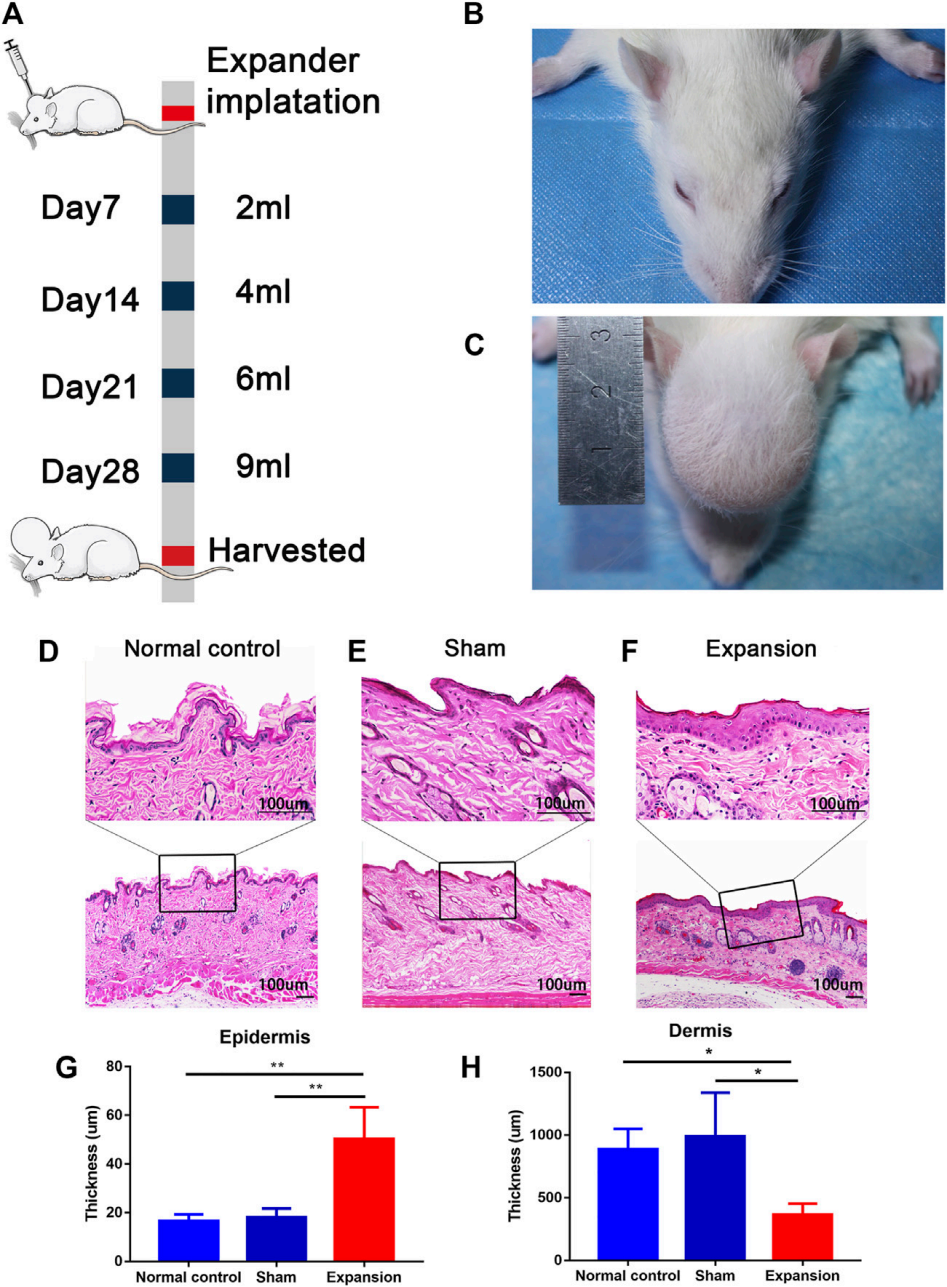
Overview of the study design and histological observation corresponding with morphological change of skin induced by mechanical stretch. **(A)** Schematic presentation of procedural timeline and expansion volume of scalp skin expansion. **(B–C)** Representative image of samples located on top of the dome induced by the skin expander and the central region of scalp in the sham operated and normal control group. **(D–H)** H and E staining showed a thicker epidermis and a thinner dermis thickness in the expanded group (*n* = 4) than that in the normal control group (*n* = 3) and the sham operated group (*n* = 3). scale bar: 100 um *p < 0.05, **p < 0.01.

We constructed a male rat scalp expansion model to evaluate the effects of mechanical stretch on skin (Figures 1A–C). H and E staining of the expanded skin showed that the expanded group had a thicker epidermis and thinner dermis than the sham operated and normal control groups. There was no significant difference in the thickness of epidermis and dermis between the sham operated and normal control groups (Figures 1D–H). Therefore, the male rat scalp expansion model was successfully constructed. We observed typical histological changes induced by mechanical stretch, including increased epidermal thickness, and decreased dermal thickness.

### Transcriptomic Changes of Expanded Skin Tissue

Transcriptomic changes of skin regeneration were obtained in a scalp expansion rat model (Figure 1A). The skin transcriptome profile was evaluated in the expanded, sham operated, and normal control groups, the latter of which were set to control the influence of the surgical procedure and expander implantation.

**FIGURE 2:**
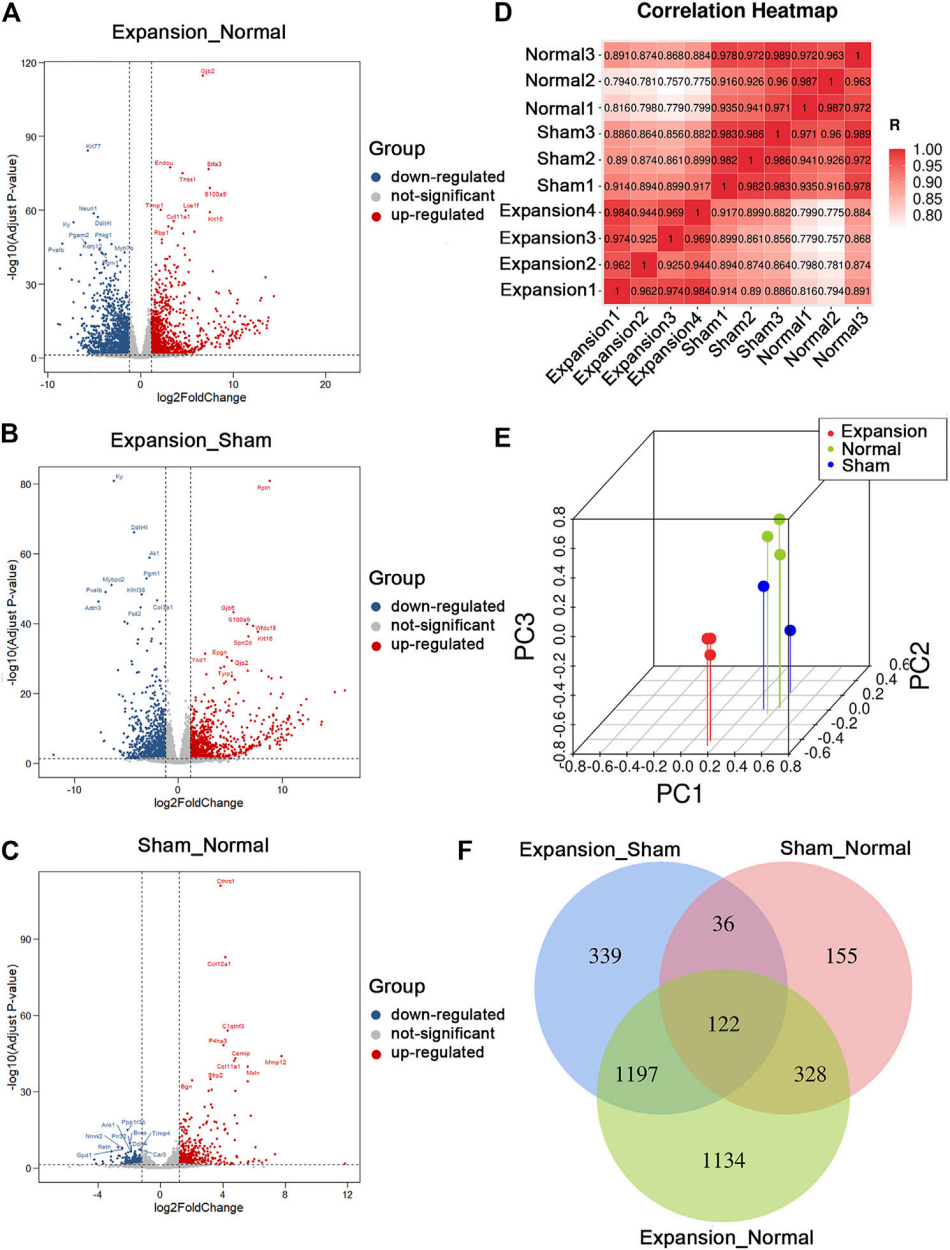
Summary of differentially expressed genes and presentation of comprehensive similarity among the expanded group, the sham operated group, and the normal control group. **(A)** Analysis of differentially expressed genes between the expanded group and the normal control group by volcano mapping. **(B)** Analysis of differentially expressed genes between the expanded group and the sham operated group by volcano mapping. **(C)** Analysis of differentially expressed genes between the sham operated group and the normal control group by volcano mapping. **(D)** HeatMap of correlation analysis with unsupervised hierarchical clustering among the expanded, sham-operated, and normal control group. **(E)** 3D scatter of PCA among the expanded, sham operated, and normal control group. **(F)** Venn diagram showed the overlap of differentially expressed genes induced by mechanical stretch (blue + green, DEGs between the sham and normal groups were removed from those between the expanded and normal groups. The remaining DEGs were compared with those between the expanded and sham groups to obtain overlapping 1197 DEGs).

In total, we generated on average of 4.0 ± 1.8 million reads per sample. We aligned the reads to the ENSEMBL build reference genome and yielded an average mapping rate of 96 ± 0.18%. The base accuracy rate of Q30 was more than 92.96%, indicating that the sample processing and sequencing was of high quality (Supplementary Table S2). After performing data filtering, gene expression levels (RPKM), were obtained for 32,623 genes annotated in the RefSeq database. We identified 3,454 DEGs between expanded and normal skin, 2,259 DEGs between expanded and sham operated skin, and 883 DEGs between sham operated and normal skin (|log2FC| ≥ 1.2 and *p* < 0.05, q < 0.05). We identified significant changes in the expression of 1,660 upregulated genes and 1,794 downregulated genes between the expanded and normal control groups, 1,192 upregulated genes and 1,067 downregulated genes between the expanded and sham operated groups, and 548 upregulated genes and 335 downregulated genes between the sham operated and normal control groups (Figures 2A–C; Supplementary Table S3).

Unsupervised clustering revealed clear differences among the three groups. The expanded skin samples were grouped together based on the similarity of their gene expression profiles and were separated from control samples. This confirmed the uniformity within the group and the independence between groups (Figure 2D). PCA showed that mechanical stretch and foreign object implantation had profound effects on gene expression, further indicating the accuracy and reliability of the RNA-seq data (Figure 2E).

The comparative analysis of DEGs revealed that the number of DEGs between the expanded and sham groups and between the sham and normal groups was close to the number of DEGs between the expanded and normal groups. These results indicated that the molecular changes primarily resulted from mechanical stretch and foreign object implantation. To determine the genes regulated by mechanical stretch, DEGs between the sham and normal groups were removed from those between the expanded and normal groups. The remaining DEGs were compared with those between the expanded and sham groups to obtain overlapping DEGs, and 1,197 DEGs, including 663 upregulated and 534 downregulated genes, were acquired to analyze the effect of mechanical stretch (Figure 2F).

### Functional Enrichment and Pathway Analysis

**FIGURE 3:**
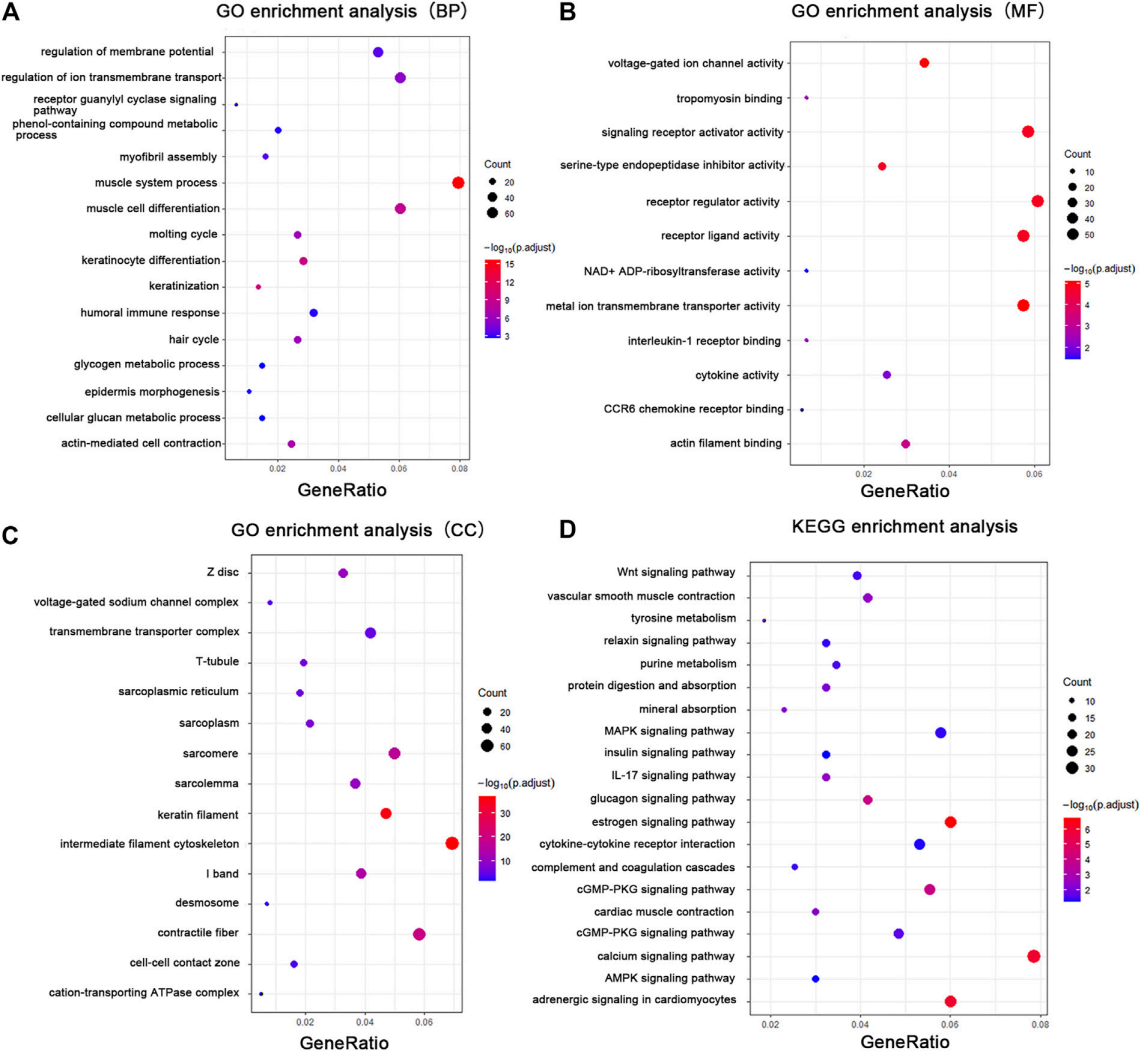
GO enrichment analysis and KEGG analysis showed changes in the molecular response and signaling pathway changes of skin during tissue expansion. **(A)** GO enrichment analysis of the DEGs in biological processes. **(B)** GO enrichment analysis of the DEGs in molecular functions. **(C)** GO enrichment analysis of the DEGs in cellular components. **(D)** KEGG analysis of the DEGs. Gene ratio indicates the number of DEGs associated with the GO term divided by the total number of DEGs. The size of the dots represents the number of DEGs associated with the GO term and the color represents the negative value of log_10_ of adjusted p-value. BP biological processes, MF molecular functions, CC cellular components.

To elucidate the relationship between mechanical stretch and skin growth, functional enrichment analysis was performed on the identified set of DEGs. These findings revealed significant enrichment in various biological processes, primarily in muscle system process (GO:0003012), keratinocyte differentiation (GO:0030216), actin-mediated cell contraction (GO:0070252), hair cycle (GO:0042633), regulation of ion transmembrane transport (GO:0034765), myofibril assembly (GO:0030239), epidermis morphogenesis (GO:0048730), and glycogen metabolic process (GO:0005977) (Figure 3A; Supplementary Table S4). Consistent with the biological process, the cellular component included intermediate filament (GO:0045111), contractile fiber (GO:0043292), keratin filament (GO:0045095), transmembrane transporter complex (GO:1902495), cell–cell contact zone (GO:0044291), and desmosome (GO:0030057) (Figure 3B; Supplementary Table S5). The enrichment analysis showed metal ion transmembrane transporter activity (GO:0046873), receptor ligand activity (GO:0048018), voltage-gated ion channel activity (GO:0005244), signaling receptor activator activity (GO:0030546), tropomyosin binding (GO:0005523), and cytokine activity (GO:0005125) (Figure 3C; Supplementary Table S6) in the molecular function category.

Pathway analysis was performed with the KEGG Mapper search pathway tool to investigate the molecular pathways affected by mechanical stretch. The analysis showed that 11 pathways were involved, including the estrogen signaling pathway (rno04915), adrenergic signaling in cardiomyocytes (rno04261), calcium signaling pathway (rno04020), glucagon signaling pathway (rno04922), cGMP-PKG signaling pathway (rno04022), MAPK signaling pathway (rno04010), IL-17 signaling pathway (rno04657), cytokine–cytokine receptor interaction (rno04060), Wnt signaling pathway (rno04310), vascular smooth muscle contraction (rno04270), and mineral absorption (rno04978) (Figure 3D; Supplementary Table S7).

In addition, the H, C2, and C5 collections were used to identify significant gene sets associated with mechanical stretch during tissue expansion through GSEA. The results revealed that molecular alterations in the skin induced by mechanical stretch were associated with muscle system process, actin-mediated cell contraction, epidermis development, and cellular glucan metabolic process (Supplementary Figure S1). Furthermore, the enriched gene sets were somewhat similar to those shown in GO analysis, as shown in Supplementary Table S8.

### PPI Network Analysis

The PPI network was constructed using the STRING web tool and visualized in Cytoscape. PPI network analysis obtained 460 nodes and 1,236 protein interaction pairs. Significant gene modules were identified as clusters 1–7 by using the MCODE application in Cytoscape (Supplementary Table S9). Enrichment analysis via ClueGO was used to determine the functions of clusters.

**FIGURE 4:**
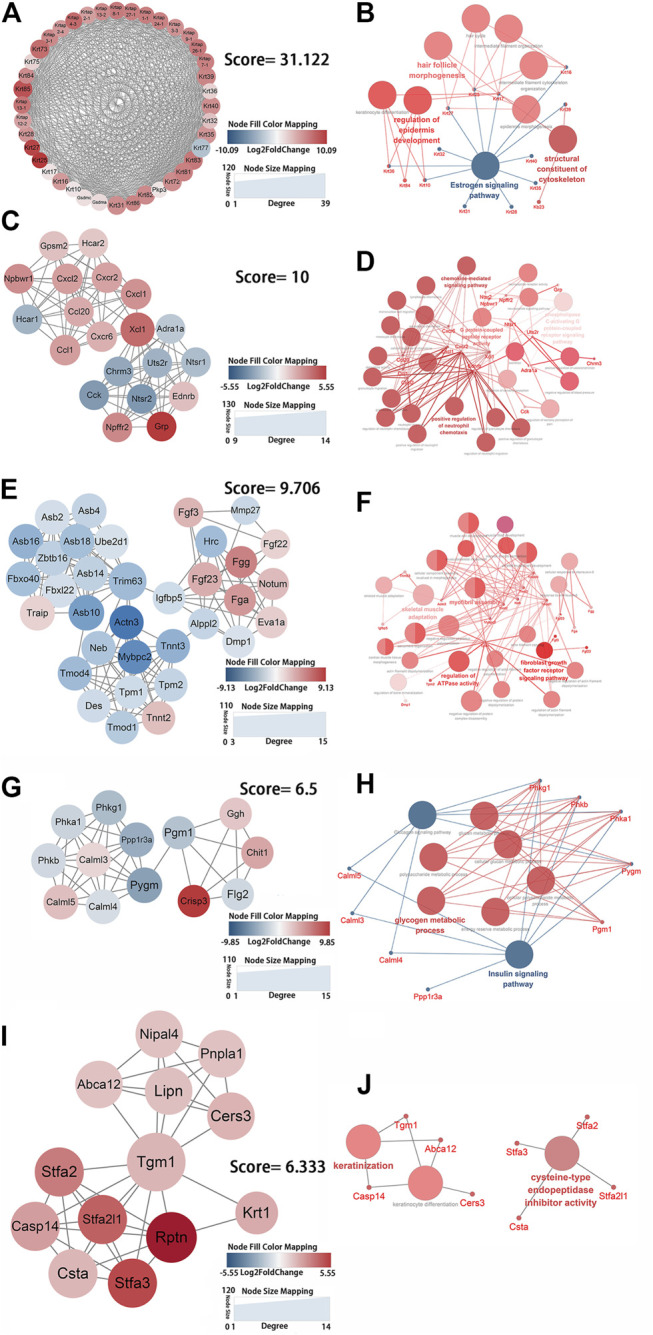
Identification of the key protein clusters using string database and MCODE plugin (nodes >10, score >5) and functional analysis of identified 1-5 key protein clusters using ClueGo plugin. **(A)** Color coded network of protein cluster 1 and their connection. **(B)** Bubble plot of functional enrichment analysis showing proteins in cluster 1 participated in a structural constituent of keratin filament and intermediate filament cytoskeleton. **(C)** Color coded network of protein cluster 2 and their connection. **(D)** Bubble plot of functional enrichment analysis showing proteins in cluster 2 participated in chemokine signaling pathway. **(E)** Color coded network of protein cluster 3 and their connection. **(F)** Bubble plot of functional enrichment analysis showing proteins in cluster 3 participated in myofibril assembly. **(G)** Color coded network of protein cluster 4 and their connection. **(H)** Bubble plot of functional enrichment analysis showing proteins in cluster 4 participated in glycogen metabolic process **(I)** Color coded network of protein cluster 5 and their connection. **(J)** Bubble plot of functional enrichment analysis showing proteins in cluster 5 participated in keratinocyte differentiation. The nodes in **(A) (C) (E) (G) (I)** indicate proteins. The edges represent protein interaction. The bubbles in **(B) (D) (F) (H) (J)** indicate GO term, the dots represent proteins, and the edges represent the proteins enriched in GO term.

Genes in cluster one were keratins and keratin-associated proteins, which were present in keratin filaments and intermediate filaments in the cytoskeleton. These genes were significantly enriched in intermediate filament organization (GO:0045109), regulation of epidermis development (GO:0045682), and hair follicle morphogenesis (GO:0031069) according to biological process analysis and in the estrogen signaling pathway (KEGG:04915) according to KEGG pathway analysis (Figures 4A,B; Supplementary Table S10). Cluster two contained genes that entailed G protein-coupled peptide receptor activity (GO:0008528) and the chemokine-mediated signaling pathway (GO:0070098) (Figures 4C,D; Supplementary Table S11). The enriched genes from cluster three were mainly in myofibril assembly (GO:0030239) (Figures 4E,F; Supplementary Table S12). Cluster four was comprised of genes that were involved in glycogen metabolic processes (GO:0005977) and the glucagon signaling pathway (KEGG:04922) (Figures 4F,G; Supplementary Table S13). Genes in cluster five were enriched in keratinocyte differentiation (GO:0030216) (Figures 4I,J). The functional enrichment analysis showed that genes in cluster six had a primary role in the positive regulation of IκB kinase/NF-κB signaling (GO:0043123) of the JNK cascade (GO:0046330) (Supplementary Figures S2A,B). Fractional genes of cluster seven were related to the voltage-gated sodium channel complex and actin-mediated cell contraction (Supplementary Figures S2C,D). Seven gene modules demonstrated remarkable roles in the positive regulation of epidermis development and keratinocyte differentiation, the chemokine signaling pathway, myofibril assembly, glycogen metabolic process, positive regulation of IκB kinase/NF-κB and JNK cascade signaling, and negative regulation of voltage-gated sodium channel activity. These changes can be considered key processes that are activated by mechanical stretch.

**FIGURE 5:**
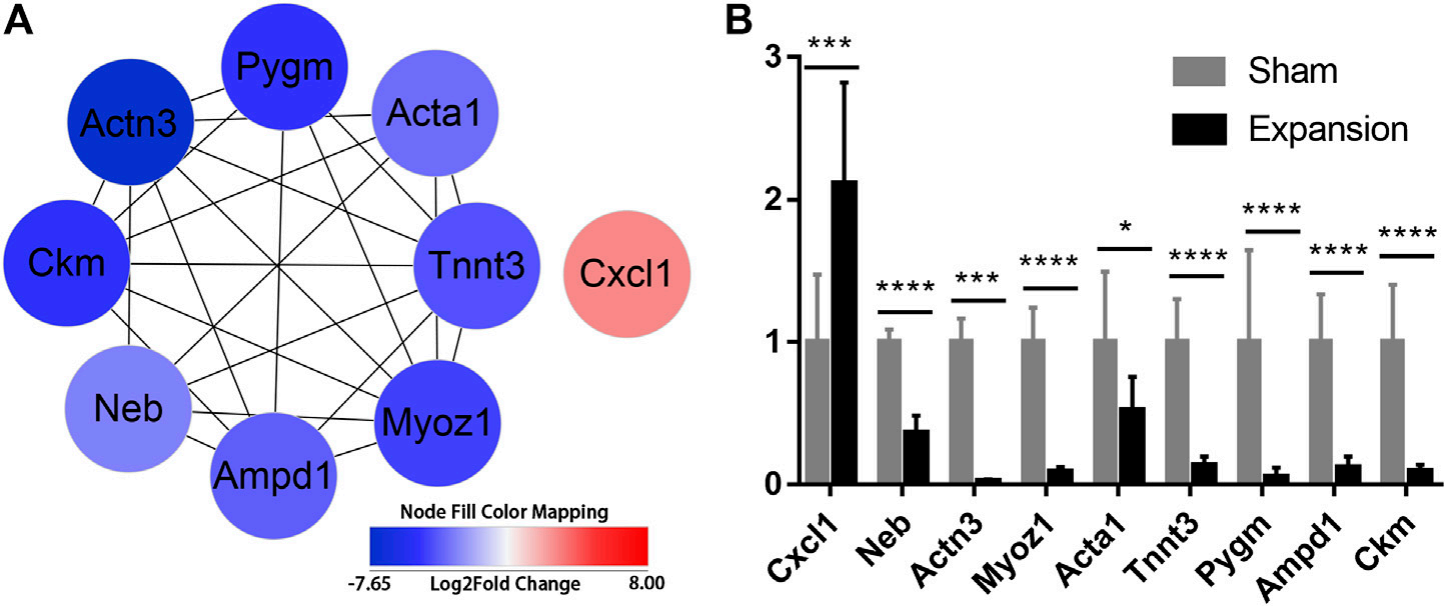
Identification of hub genes activated by mechanical stretch and their expression confirmed by qPCR **(A)** Hub genes identified with PPI analysis **(B)** The relative mRNA levels of CXCL1, NEB, ACTN3, MYOZ1, ACTA1, TNNT3, PYGM, AMPD1, and CKM using qPCR. *p < 0.05, ***p < 0.001, ****p < 0.0001.

To further excavate the hub genes activated by mechanical stretch in tissue expansion, CytoHubba was used. CXCL1, NEB, ACTN3, MYOZ1, ACTA1, TNNT3, PYGM, AMPD1, and CKM were identified as the hub genes, which were ranked by their degree of centrality (Figure 5A; Supplementary Table S14). Functional analysis revealed that these genes play a crucial role in cytoskeleton remodeling, the alteration of metabolic processes, and the positive regulation of leukocyte chemotaxis. The expression of nine genes was confirmed, and the results of RNA-seq and q-PCR revealed a similar trend (Figure 5B). The results suggested that the RNA-seq data were reliable and that the above genes are involved in the molecular alterations in the skin induced by mechanical stretch.

### TF–mRNA Interaction Regulatory Network Construction

**FIGURE 6:**
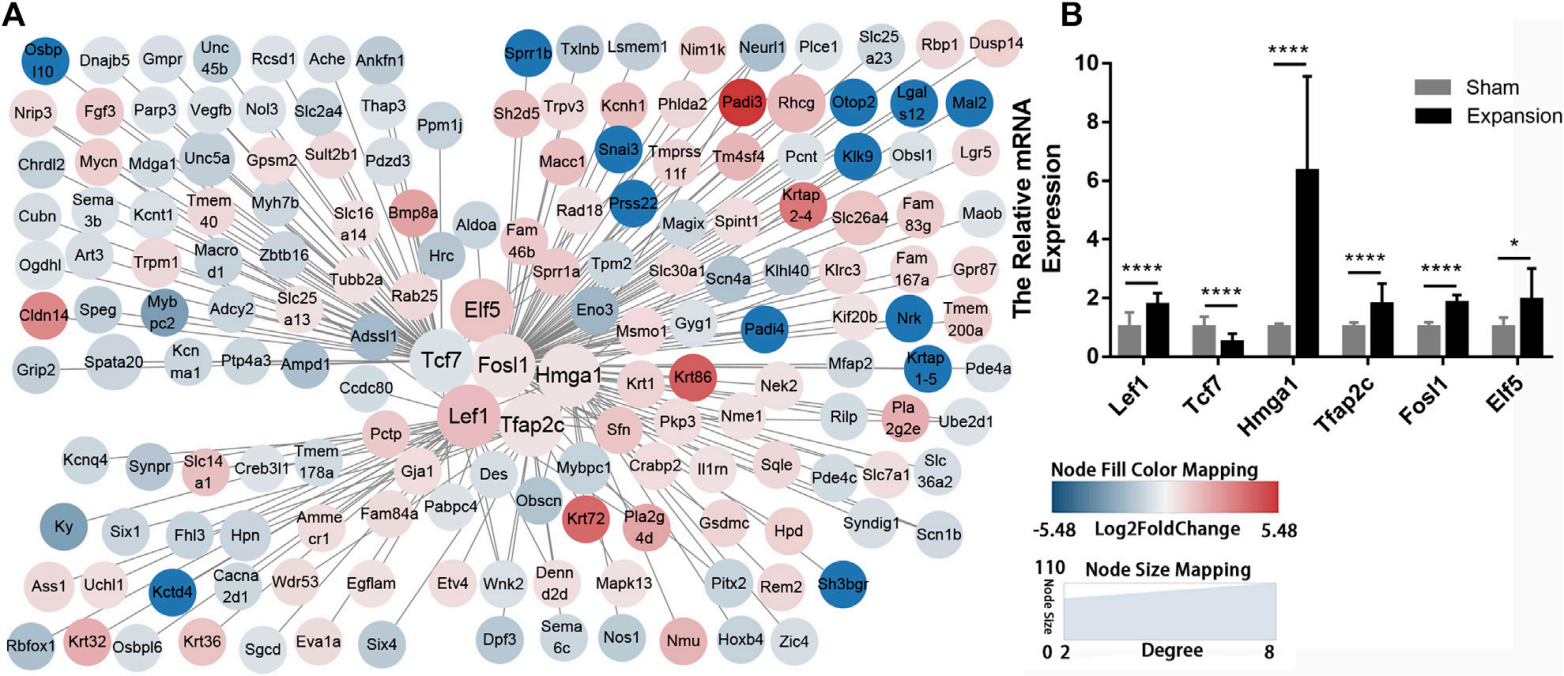
Construction of regulatory networks of TF-mRNA interactions using transcription factor databases **(A)** Regulatory networks of TF-mRNA interactions activated by mechanical stretch **(B)** The relative mRNA levels of LEF1, TCF7, ELF5, FOSL1, TFAP2C, and HMGA1 using qPCR. *p < 0.05, ****p < 0.0001.

TFs are proteins that coordinate gene expression in a spatial and temporal manner in specific cell types. These proteins are involved in transcriptional regulation and directly shape organism phenotypes by inducing or maintaining the cell fate in response to abiotic stimulus. We identified six TFs (LEF1, TCF7, HMGA1, TFAP2C, FOSL1, and ELF5) and explored their target genes by employing a relative database (details in Methods). Overlapping mRNAs between the targets of TFs and DEGs are summarized in Supplementary Table S15. TF–mRNA interaction regulatory networks were constructed and visualized by Cytoscape (Figure 6A). The results showed that the expression of the six TFs were in accordance with the results of RNA-seq (Figure 6B). The TF–mRNA network predicts the potential mechanism by which TFs carry out their activity, which can facilitate subsequent experimental verification.

## Discussion

Tissue expanders are frequently used worldwide for skin regeneration and reconstruction. Skin soft tissue expansion has several advantages; the extra skin is from the same area and matches its color, texture, and thickness, which are valuable features for reconstruction. However, the capacity for skin expansion is limited, and the molecular changes and biological processes in skin that are provoked by mechanical stretch remain unclear. Using bioinformatics approaches, we comprehensively investigated gene expression patterns evident in skin induced by mechanical stretch and identified nine robust hub genes (CXCL1, NEB, ACTN3, MYOZ1, ACTA1, TNNT3, PYGM, AMPD1, and CKM) that may serve as key molecules in skin growth. The genes were shown to be involved in several important biological processes, including keratinocyte differentiation, cytoskeleton reorganization, the chemokine signaling pathway, glycogen metabolism, and voltage-gated ion channel activity. We also identified potentially significant pathways, including the Wnt signaling pathway, the glucagon signaling pathway and cytokine–cytokine receptor interaction. In addition, we identified six TFs (LEF1, TCF7, HMGA1, TFAP2C, FOSL1, and ELF5) and constructed TF–mRNA interaction regulatory networks. Therefore, our results provide significant insight into the process of skin expansion induced by mechanical stretch.

The hub genes were identified based on topological analysis of the PPI by CytoHubba and may serve as key molecules in promoting skin regeneration during tissue expansion. These hub genes include the upregulated gene CXCL1 and the downregulated genes NEB, ACTN3, PYGM, MYOZ1, ACTA1, AMPD1, TNNT3, and CKM. The hub proteins are associated with several different biological processes. C-X-C motif chemokine ligand 1 (CXCL1) plays a role in the inflammatory response and as a chemoattractant for neutrophils. Mechanical stretch increases the expression of CXCL1 in skin cells, as well as in liver sinusoidal endothelial cells and vascular smooth muscle cells (Zhao et al., 2017; Hilscher et al., 2019). CXCL1 can stimulate keratinocyte migration and/or proliferation and is secreted by keratinocytes during re-epithelialization (Kroeze et al., 2012). Furthermore, CXCL1 has an important function in angiogenesis via CXCR2 (Addison et al., 2000). These studies indicate that CXCL1 might be also involved in mechanical stretch-induced keratinocyte proliferation and migration during tissue expansion. Furthermore, tissue expansion involves the maintenance of chronic inflammation, and tissue regeneration was associated with lower concentrations of many different inflammatory markers (Ding et al., 2019). However, blocking inflammation by dexamethasone administration did not decrease the proliferation of skin cells (Aragona et al., 2020). Taken together, mechanical stretch induces inflammation-related gene expression (including CXCL1, CXCL2, CXCR2, CCL20, and CCR6), which may play important roles in the stress response against tissue expansion.

Downregulation of the hub protein nebulin (NEB) greatly increases thin filament extensibility and impairs thin filament configuration (Kiss et al., 2018). Downregulated NEB results in reduced contractile strength and diminished force generation of cells; as such, the skin may maintain microenvironmental homeostasis when experiencing external mechanical stretch during tissue expansion. Actinin alpha 3 (ACTN3) is a widely expressed cytoskeleton protein that crosslinks actin-containing thin filaments, which connect epithelial adherens junctions with focal adhesions at the leading edge of migrating cells (Izdebska et al., 2020). ACTN3-related pathways have been implicated in muscle contraction and the regulation of cytoskeleton remodeling. In addition, ACTN3 deficiency reduces glycogen phosphorylase activity and results in a fundamental shift toward more oxidative pathways of energy utilization (Berman and North, 2010). Therefore, ACTN3 is not only involved in cytoskeleton remodeling by regulating the actin cytoskeleton via Rho GTPases, but also in metabolic alteration provoked by mechanical stress. Myozenin 1 (MYOZ1) plays a role in the negative regulation of phosphoprotein phosphatase activity, while decreased MYOZ1 expression results in enhanced calcineurin signaling (Frey et al., 2008). PPI analysis indicates that MYOZ1 can interact with filamin c (FLNC), LIM domain binding 3 (LDB3), ACTN3, and troponin T3 (TNNT3). Transcriptomic analysis shows FLNC, LDB3, ACTN3, and TNNT3 are downregulated (Supplementary Figure S3). Moreover, FLNC participates in the anchoring of membrane proteins for the actin cytoskeleton. LDB3 couples protein kinase C-mediated signaling to the cytoskeleton and functions as an adaptor in cytoskeletal assembly. Actin alpha 1, skeletal muscle (ACTA1) belongs to the actin family of proteins, which are highly conserved proteins that play a role in cell motility, structure, and integrity. Decreased ACTA1 expression can contribute to muscle weakness (Joureau et al., 2018), while troponin T3 (TNNT3) and ACTA1 have a collaborative function in muscle contraction (Wei and Jin, 2016).

Glycogen phosphorylase, muscle associated (PYGM) is a key enzyme that regulate glucose homeostasis and energy metabolism plasticity. The two mechanisms of PYGM activation comprise reversible phosphorylation and allosteric regulation. Specifically, GPCRs–AC–cAMP–PKA–PK is considered the classical pathway leading to PYGM activation (Llavero et al., 2019). In addition, different isoforms of AC can be activated by both Ca^2+^ and direct phosphorylation due to the action of PKA, PKC, or Raf-1 (Beazely et al., 2005). Further, increased Ca^2+^ in the cytosol and PKA, PKC, or Raf-1 activation can be induced by mechanical stretch and may be a mechanism for PYGM phosphorylation. However, the PYGM phosphorylation status that influences glucose metabolism during tissue expansion requires further experimental study. Adenosine monophosphate deaminase 1 (AMPD1) plays a critical role in the purine nucleotide cycle and energy metabolism. Creatine kinase, M-type (CKM) is a cytoplasmic enzyme involved in energy homeostasis; it reversibly catalyzes the transfer of phosphate between ATP and various phosphogens, such as creatine phosphate (Wyss and Kaddurah-Daouk, 2000). In this study, the downregulated hub genes were mainly involved in cytoskeleton remodeling and the alteration of metabolic processes induced by mechanical stretch.

The STRING database and MCODE computational methods were used to understand the biological processes of mechanical stretch-induced alterations in gene expression by the skin, in addition to the potential regulatory mechanism of skin regeneration. Keratins (KRTs) are an interesting set of molecules in cluster one that are increasingly recognized as being involved in cell regulation and signal transduction, in addition to their central structural role as cell stress protectors. Consistent with the keratinocyte proliferation and differentiation observed at the histological level, keratins are significantly upregulated at the molecular level affected by mechanical stretch. It is widely accepted that KRT17 serves as a molecular marker for hyperproliferative keratinocytes; it may confer upon activated keratinocytes a moderate level of mechanical scaffolding and sufficient plasticity for migration and re-epithelialization. Furthermore, KRT17 is involved not only in regenerating and migrating epidermal keratinocytes after skin injury, but also in influencing cell growth and size of keratinocytes by regulating the cell cycle. Additionally, the colocalization of KRT17 with Aire protein can amplify inflammatory and immune responses in diseased epithelia (Hobbs et al., 2015). Interestingly, it has been reported that KRT17 was released from intermediate filaments and translocated into the nucleus via a nuclear localization signal (NLS), thereby mediating cell cycle progression and tumor growth (Escobar-Hoyos et al., 2015). KRT10 is a carefully regulated differentiation-specific keratin protein. KRT10 plays extra functional roles in inhibiting keratinocyte proliferation and cell cycle progression, and loss of KRT10 leads to increased keratinocyte turnover. Furthermore, KRT5 expression induced by progestins was sufficient to promote cell junction reconstruction and cell morphology remodeling in breast cancer (McGinn et al., 2020). Herein, our data suggest that KRTs might be switched on after mechanical stretch and play an essential role in cytoskeleton reconstruction, thereby promoting keratinocyte proliferation and immune responses.

The common functions of cluster two were mainly linked to the chemokine-mediated signaling pathway. As previously reported, cytokines were upregulated in keratinocytes under mechanical stretch both *in vivo* and *in vitro*. These indicated that the inflammatory response was strongly activated when mechanical stretch was applied (Qiao et al., 2019). However, a severe inflammatory response leads to skin ulceration and exposure of the tissue expander. Skin undergoing stretch and relaxation is generally expected to experience injury and repair. Moreover, a controllable, moderate inflammatory response plays a significant role in promoting keratinocyte proliferation and initiating skin growth (Ledwon et al., 2020). Chemokines then act on seven-transmembrane G-protein-coupled receptors and are characterized as leukocyte chemotactic signals, which exhibit differential chemotactic activity for different classes of leukocytes. Both CCL20 and its receptor CCR6 are upregulated in skin that has undergone mechanical stretch. CCL20 has been previously reported to regulate macrophage recruitment and facilitate M0 macrophage polarization toward M2 macrophages (Argyle and Kitamura, 2018; Wang et al., 2020c). Furthermore, our previous study revealed a substantially higher density of M2 macrophages than M1 macrophages during tissue expansion (Ding et al., 2019). Thus, these findings suggested that mechanical stretch may modulate CCL20 secretion to induce M2 macrophage polarization and promote skin regeneration. CCL20 expression is also elevated in many cancers. Thus, cancer cells can stimulate their own proliferation and migration in an autocrine manner, as well as cause angiogenesis via the binding of CCL20 to CCR6 (Wang et al., 2016; Zhu et al., 2018). Moreover, the expression of CXCL1, CXCL2, and their common receptor CXCR2 are increased in expanded skin, and CXCL1 is the sole upregulated hub gene. Tissue expansion has been shown to induce hypoxia in the overlying skin. Studies indicated that chronic hypoxia increases CXCL1 and CXCL2 expression in macrophages (Fang et al., 2009). In addition, CXCL1 and CXCL2 play a significant role in boosting cancer cell proliferation and migration; CXCL1 also contributes to stemness and regulating epithelial-mesenchymal transition (EMT)-related processes (Wang N. et al., 2017; Valeta-Magara et al., 2019). Chemokines can recruit leukocytes to stimulate angiogenesis and re-epithelialization; therefore, determining the underlying functions of chemokines during tissue expansion and contriving ways to interfere with signaling are likely to have great potential for modulating inflammation in expanded skin and facilitating skin regeneration.

The biological processes of cluster three primarily relate to myofibril assembly, which is defined as myofibril formation and may be involved in cytoskeleton remodeling. The structure and distribution of the cytoskeleton is profoundly influenced by mechanical stretch. The rearrangement of actin organization and remodeling at adherens junctions are essential to mediate responses to mechanical stretch, such as cell proliferation (Aragona et al., 2020). For instance, DIAPH3 is a member of the formin family, which is involved in actin remodeling, and MYH9 is a key subunit of myosin IIA; the depletion of DIAPH3 and MYH9 results in no increase in cell proliferation following mechanical stretch. Herein, many genes controlling cell cytoskeleton and actin filament organization, including actin filament organization regulators (NEB and MYBPC2) and actin filament capping (TPM1, TPM2, TMOD1, and TMOD4), were preferentially downregulated during tissue expansion. Meanwhile, some genes related to actin crosslink formation (TNNT2) were shown to be upregulated. TPM1, TPM2, and TNNT3 have been previously shown to be downregulated during tissue expansion (Ledwon et al., 2020). These genes may control the morphology and polarity of the cell during tissue expansion. NEB is a giant protein that spans the actin filament in skeletal muscle. It is reported that conditional knockout (NEB cKO) undermines thin filaments of skeletal muscle and impairs tropomyosin (Tpm) and troponin (Tnnt) movement, thereby negatively affecting thin filament activation and cross-bridge recruitment. Therefore, we propose that the downregulation of NEB caused by mechanical stretch impairs TNNT2, TNNT3, and TPM1 expression to weaken contractile thin filaments and adapt to external stretch change. As key components of contractile stress fibers in nonmuscle cells, TMOD1 and TMOD4 bind actin-Tpm filaments to protect them from depolymerizing, not elongating. Thus, TMODs are essential for the maintenance of contractile actomyosin bundles. Collectively, myofibril assembly is critical for the regulation and maintenance of cytoskeleton remodeling in response to external mechanical stretch stimuli.

GO analysis of biological processes and KEGG analysis both clearly showed that most genes in cluster four are associated with glycogen metabolism. Metabolic pathways can control the proliferation and differentiation of skin cells, especially epidermal cells with active metabolism. Phosphorylase kinase, a multimeric enzyme with four subunits (α, β, γ, and δ), integrates multiple calcium/calmodulin-dependent signaling pathways while coupling them glycogenolysis and ATP-dependent phosphorylation. This process ensures a continuing energy supply for activities such as cell migration and proliferation. Phosphorylase kinase catalytic subunit gamma 1 (PHKG1), phosphorylase kinase regulatory subunit alpha 1 (PHKA1), phosphorylase kinase regulatory subunit beta (PHKB), and PYGM are downregulated under conditions of mechanical stretch. The phosphorylase kinase is responsible for PYGM phosphorylation; their downregulation indicates that mechanical stretch affects the metabolism of the expanded skin, which mainly reflects the inhibition of glycogenolysis. To our knowledge, mechanical stretch often induces hypoxia in expanded skin (Liang et al., 2014). Furthermore, epidermal cellular metabolism presents a biphasic glycogen utilization pattern, with initial depletion followed by increased glycogen content in response to limited local oxygen levels (Straseski et al., 2009). A recent study showed that glycolysis responds to architectural features of the actomyosin cytoskeleton, which couple cancer cell metabolism to the mechanical properties of the surrounding tissue (Park et al., 2020). Another transcriptomic analysis related to mechanical stretch-induced skin changes in pigs showed the involvement of an expansive list of metabolic processes, including glycolysis (Ledwon et al., 2020). However, in this study, the results of KEGG analysis did not display a significant difference in glycolysis. Thus, further investigation of the alteration of oxidative phosphorylation and glycolysis induced by mechanical stretch is required.

We also studied TF–mRNA interactions to identify the transcriptional regulators of the identified DEGs. As such, we identified six TFs as regulators of the DEGs in mechanically stretched skin. Both transcription factor 7 (TCF-7) and lymphoid enhancer binding factor 1 (LEF1) are members of the T cell factor/lymphoid enhancer-binding factor family of high mobility group (HMG) box transcriptional activators. LEF1, a proxy for active Wnt signaling, is activated not only by cyclic strain and physiological tensile strain, but also in tissue expansion (Thomas et al., 2011; Luo et al., 2019). The LEF1 and Wnt signaling pathways are involved in hair cell differentiation and follicle morphogenesis (Lien et al., 2014; Adam et al., 2018). Moreover, our previous work observed that transplanted hair follicle bulge-derived stem cells (HFBSCs) contributed to tissue regeneration by differentiating into epidermal cells, vascular endothelial cells, and the outer root sheath cells of hair follicles during tissue expansion (Cheng et al., 2020). Interestingly, LEF1 is expressed in embryonic and neonatal papillary fibroblasts but is lost in adult fibroblasts (Driskell et al., 2013; Philippeos et al., 2018). Inducing LEF1 expression in adult skin fibroblasts reportedly enhances skin regeneration, including hair follicle neogenesis and arrector pili reformation in the wound (Phan et al., 2020). Our results suggest that mechanical stretch induces LEF1 expression and may play roles in skin regeneration during tissue expansion. TCF7, an active transcription factor associated with the Wnt mediator β-catenin, was induced by basic fibroblast growth factor (bFGF) treatment in fibroblasts (Wang X. et al., 2017). TCF-7 plays an important regulatory role in mature naive CD4^+^ and CD8^+^ T cell differentiation, which might participate in the immune response during tissue expansion (Zhang et al., 2021).

High mobility group AT-hook 1 (HMGA1), a nonhistone architectural chromosomal protein, widens or “opens” the minor groove of A + T rich regions in double-stranded DNA after displacing histone H1. This process facilitates the recruitment of transcription factor complexes and chromatin modifiers to modulate gene expression (Reeves and Beckerbauer, 2001). HMGA1 is highly expressed in embryonic and adult stem cells, whereas HMGA1 is low or undetectable in fully differentiated or nondividing adult cells. Similarly, we observed that HMGA1 expression is upregulated in skin exposed to mechanical stretch. Many reports have demonstrated the role of HMGA1 in regulating normal cell proliferation, embryonic cell growth and differentiation, and mesenchymal cell function and regenerative function (Reeves, 2001; Sgarra et al., 2010). *HMGA1* expression is regulated by a large number of other biological, environmental, and transcription factors, including transforming growth factor α (TGF-α), platelet-derived growth factor (PDGF), fibroblast growth factor (FGF), calcium ionophores, hypoxia, and the transcription factor AP-1 (Reeves, 2001). Moreover, HMGA1 amplifies the Wnt signaling pathway to enhance self-renewal of intestine stem cells by inducing genes expressing Wnt agonist receptors and Wnt effectors (Xian et al., 2017). In addition, HMGA1 is a downstream nuclear target of the insulin receptor (INSR) signaling pathway (Ohneda et al., 2000), which mediates insulin action and maintains glucose homeostasis (Foti et al., 2005). Furthermore, HMGA1 positively induces the expression of Forkhead box protein O1 (FOXO1) (Arcidiacono et al., 2018). FOXO1 is a master regulatory factor for gluconeogenesis and glycogenolysis and a positive regulator of the expression of insulin and a series of circulating proteins involved in glucose counter regulation (Chiefari et al., 2018). Therefore, evidence suggests that HMGA1 may induce regenerative functions via the Wnt signaling pathway and maintain glucose homeostasis.

Transcription factor AP-2 gamma (TFAP2C), a subunit of the transcription factor AP-2, is activated by nuclear envelope proteins involved in shear stress-induced vascular endothelial cell dysfunction. Our results align with those of a previous study, which showed that mechanical stretch applied to mouse skin leads to transcriptional changes in AP-2 by altering chromatin accessibility (Aragona et al., 2020). TFAP2C plays a vital role in initiating surface ectoderm differentiation, and TFAP2C-initiated progenitor cells can mature into functional keratinocytes (Li et al., 2019). Moreover, TFAP2C can promote epithelial gene expression by directly binding to their promoters during induced pluripotent stem cell (iPSC) generation from mouse fibroblasts (Wang et al., 2020a). Further studies have shown that E-cadherin (CDH1), an important downstream target of TFAP2C and a critical regulator of EMT, plays a crucial role in mechanical stretch-induced skin cell regeneration and proliferation (Huang et al., 2021).

FOS like 1 (FOSL1), a member of the FOS family, dimerizes with proteins of the JUN family and forms the transcription factor complex AP-1. Previous studies have shown that AP-1 is the most frequent motif associated with open chromatin regions following tissue expansion. Furthermore, stretch-mediated skin expansion is regulated by the EGFR–Ras–ERK pathway (Hirata et al., 2015; Yang et al., 2016). Our data and the findings of show that *in vivo* mechanical stretch induced an increase in cell proliferation and differentiation via activation of AP-1 signaling (Aragona et al., 2020). Furthermore, E74-like factor 5 (ELF5), a member of an epithelium-specific subclass of the ETS transcription factor family, regulates the later stages of terminal keratinocyte differentiation.

Though the expression and function of these six TFs have not been investigated in expanded human skin, this study will still serve as a resource of skin regeneration mediated by mechanical stretch for fundamental and clinical research. And these TFs can be potential targets for clinical therapeutics to promote skin regeneration.

Two limitations on the present study need to be acknowledged. First, although the mRNA expression of the six TFs was verified in the expanded rat scalp samples, the mRNA and protein expression of them in expanded human skin was absent. Second, all the samples were obtained from the male rat scalp tissue, it may not be able to totally reflect the effects of mechanical stretch on the regeneration of expanded female rat scalp. Despite these two limitations, this study adds to our understanding of the important biological processes and TFs in mechanically induced skin regeneration.

In conclusion, using high throughput technology and integrative analyses, the present transcriptomic study identified LEF1, TCF7, HMGA1, TFAP2C, FOSL1, and ELF5, as key TFs involved in skin regeneration induced by mechanical stretch. Several significant hub genes (CXCL1, ACTN3, PYGM, AMPD1, CKM, MYOZ1, TNNT3, NEB, and ACTA1) were identified as being related to the biological processes associated with the chemokine-mediated signaling pathway, cytoskeleton remodeling, and glycogen metabolism. Furthermore, several key genes were significantly involved in the Wnt signaling pathway, the glucagon signaling pathway, and cytokine–cytokine receptor interaction. However, these key genes and pathways still need to be verified in a large quantity of clinical specimens, at several time points. The genes must also be analyzed and validated in combination with functional verification *in vivo* and *in vitro* to finally determine the biological targets that are most beneficial for facilitating skin regeneration. The functions of these key genes and their pathways may reveal new aspects of skin regeneration under mechanical strain. Furthermore, the identified TF regulators can be used as potential candidates for clinical therapeutics and skin pretreatment before reconstructive surgery.

## Data Availability

The datasets presented in this study can be found in online repositories. The names of the repository/repositories and accession number(s) can be found below: NCBI SRA BioProject, accession no: PRJNA753961.
