# Supplementary material for: Transcriptome Profiling Reveals Important Transcription Factors and Biological Processes in Skin Regeneration Mediated by Mechanical Stretch
Source: Front Genet. 2021 Sep 29;12:757350. doi: 10.3389/fgene.2021.757350 (PMC8511326; doi:10.3389/fgene.2021.757350)
Supplement: Supplementary file 1 [file DataSheet1.zip › Data sheet 1-Supplementary_Materials/Supplementary_Figures.docx]

Supplementary Material


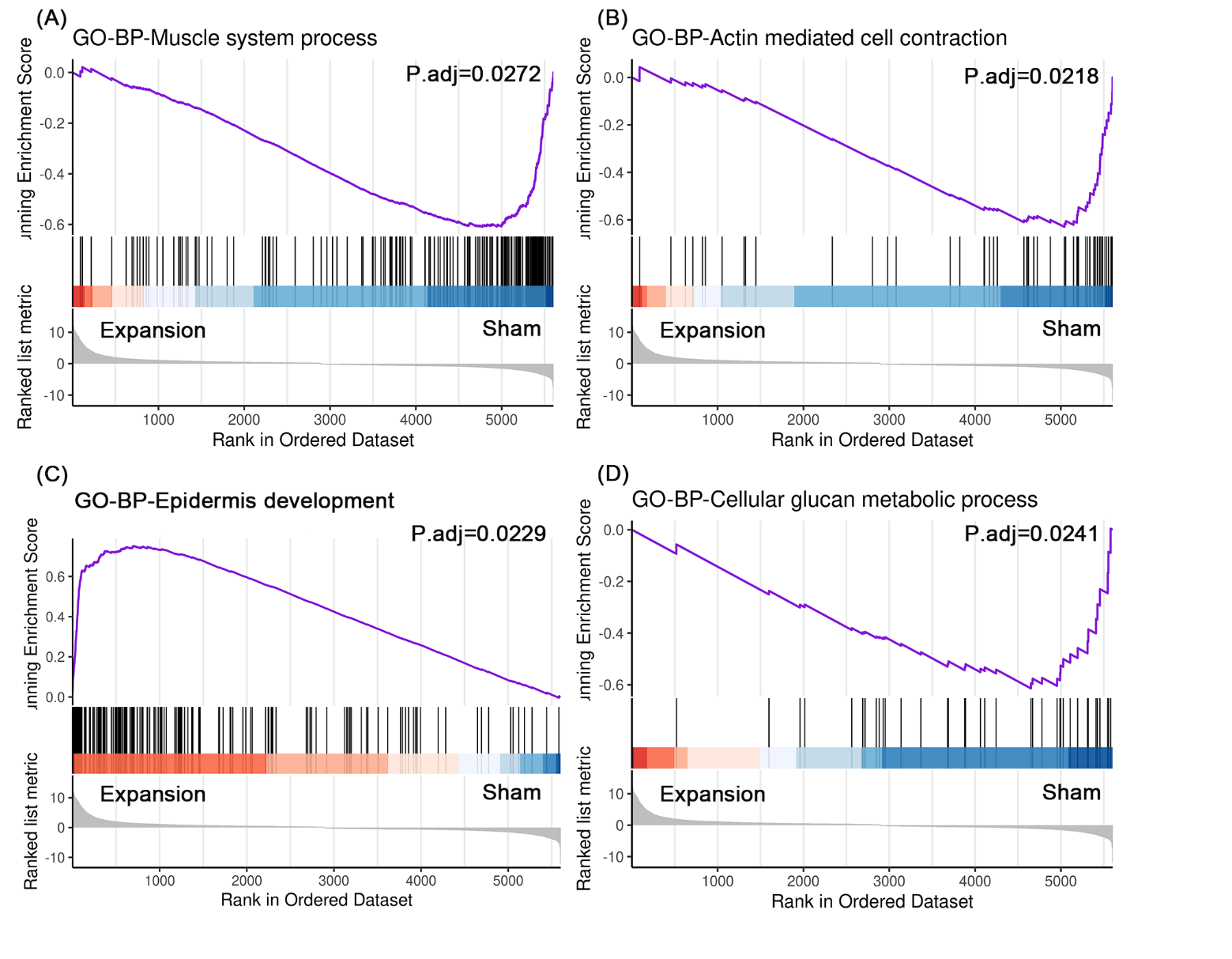


**Supplementary Figure S1.** GSEA analysis revealed related biological processes enriched in skin induced by mechanical stretch. (A)Muscle system process; (B)Actin mediated cell contraction; (C)Epidermis development; (D)Cellular glucan metabolic process..


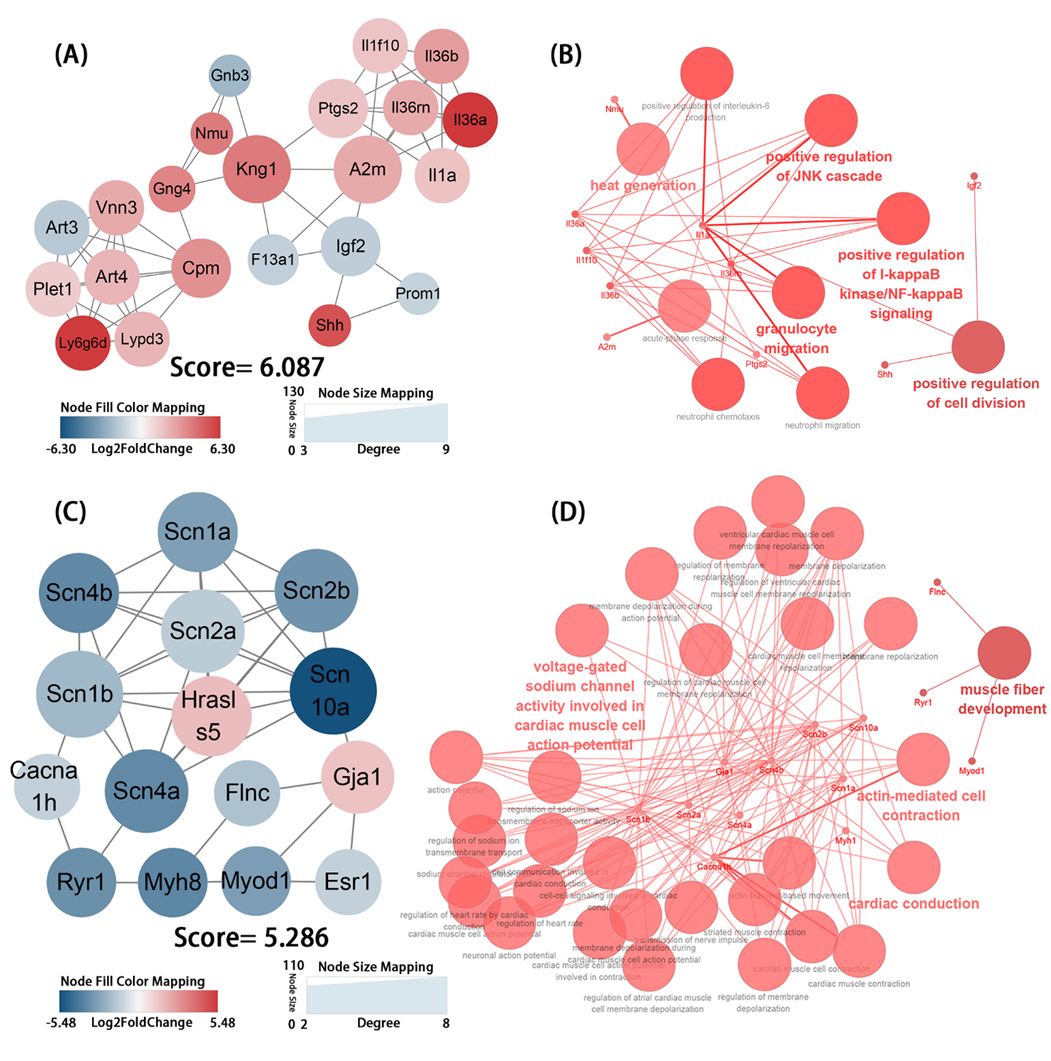


**Supplementary Figure S2.** Identification of key protein clusters using string database and MCODE plugin (nodes>10, score>5) and functional analysis of 6-7 key protein clusters identified using ClueGo plugin. (A) Color coded network of protein cluster 6 and their connection. (B) Bubble plot of functional enrichment analysis showing proteins in cluster 1 participated in positive regulation of JNK cascade. (C) Color coded network of protein cluster 7 and their connection. (D) Bubble plot of functional enrichment analysis showing proteins in cluster 2 participated in actin-mediated cell contraction.


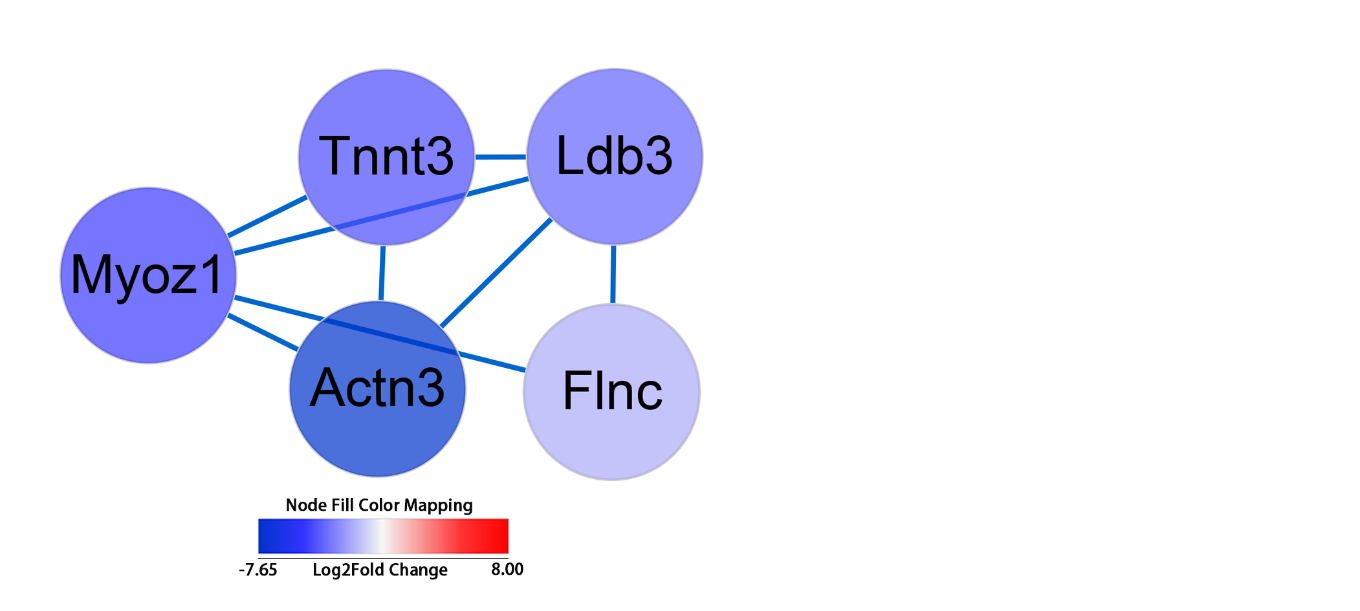


**Supplementary Figure S3.** PPI network analysis showed the interactions among MYOZ1, FLNC, LDB3, ACTN3, and TNNT3 and indicated a decrease of actin-mediated cell contraction in mechanically induced skin.
