# Supplementary figures and images for: Transcriptome Profiling Reveals Important Transcription Factors and Biological Processes in Skin Regeneration Mediated by Mechanical Stretch
Source: Front Genet. 2021 Sep 29;12:757350. doi: 10.3389/fgene.2021.757350 (PMC8511326; doi:10.3389/fgene.2021.757350)

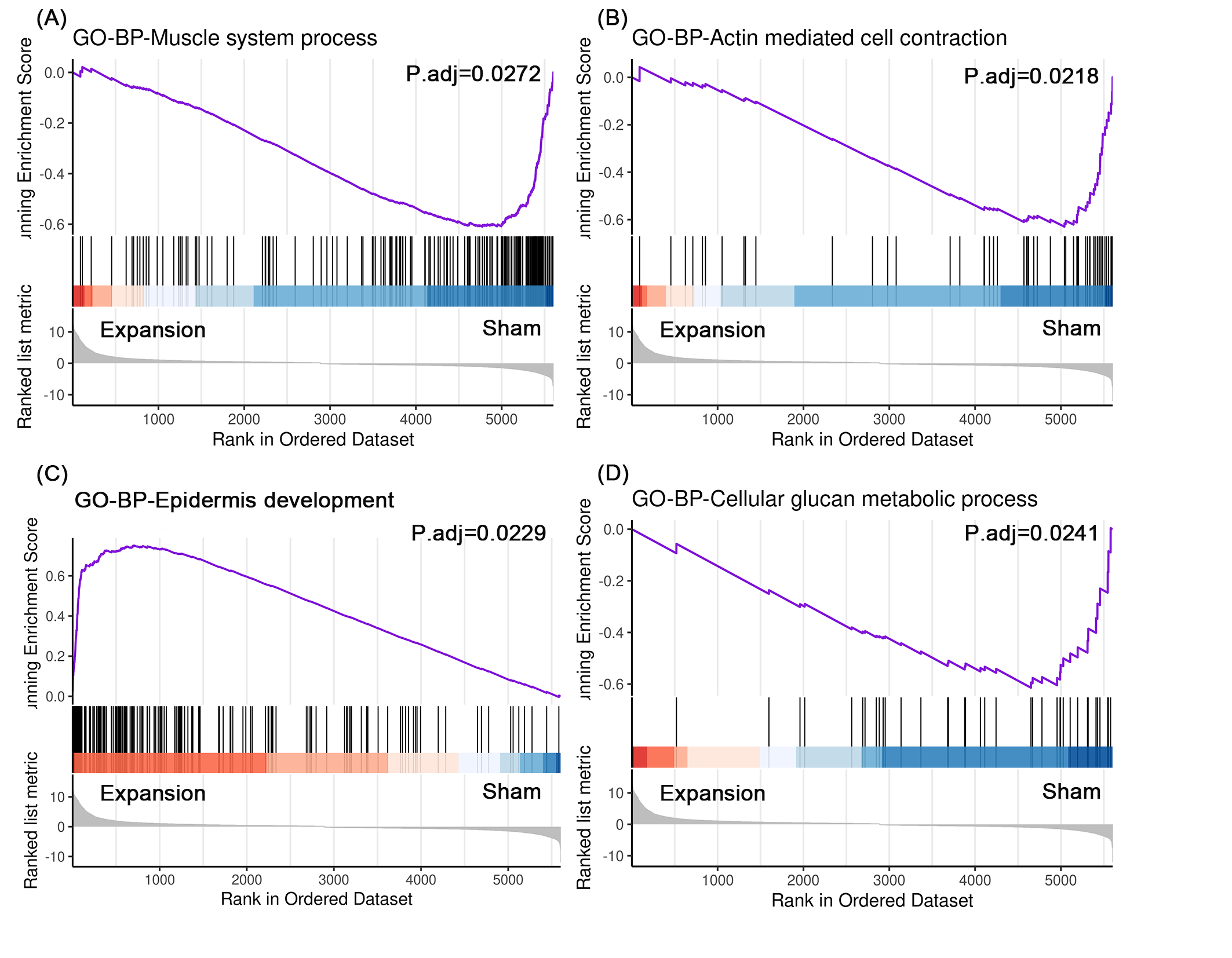

Supplement: Supplementary file 1 [file DataSheet1.zip › Data sheet 1-Supplementary_Materials/Supplementary_Figures/Supplementary Figure S1.tif]

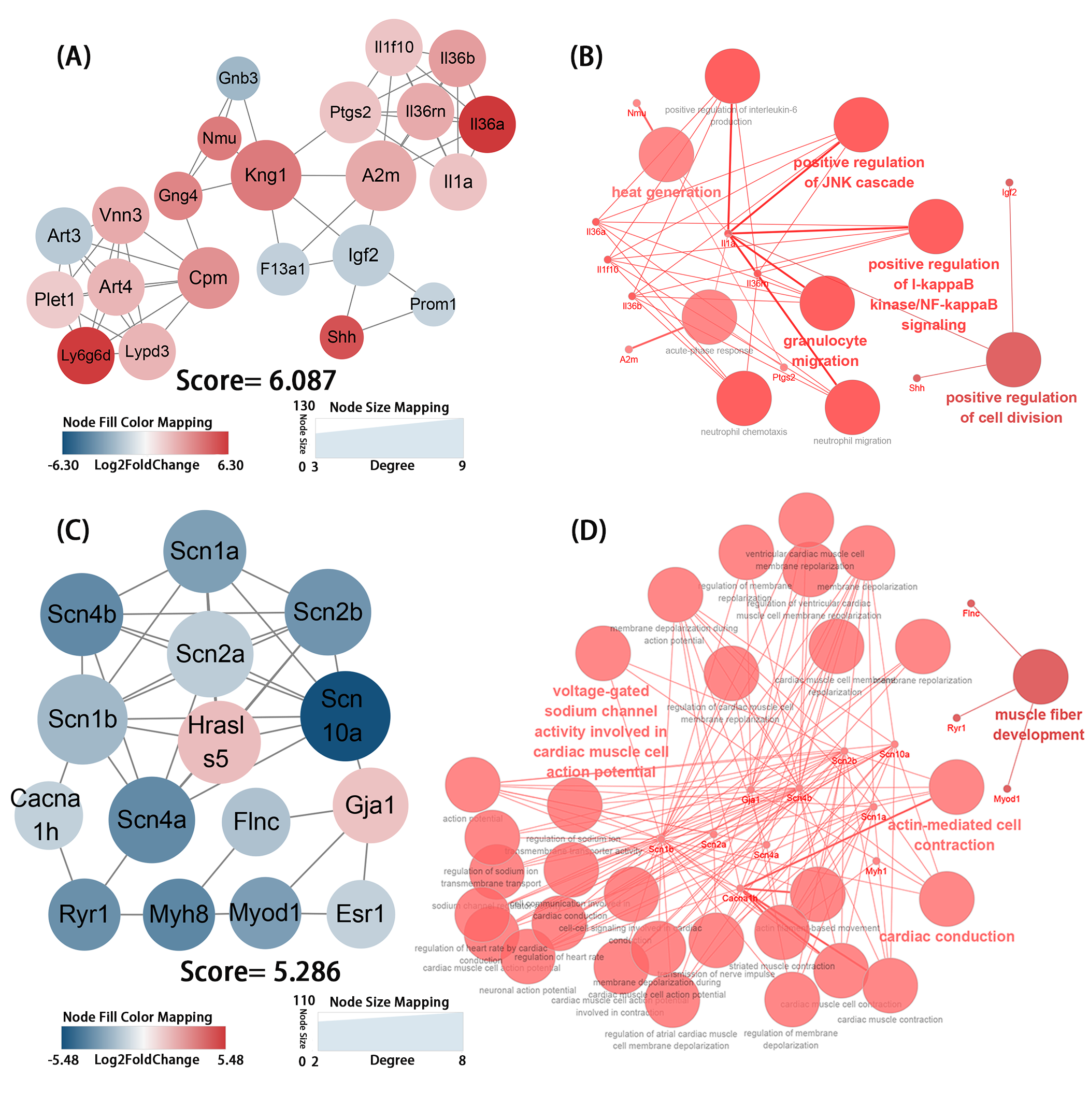

Supplement: Supplementary file 1 [file DataSheet1.zip › Data sheet 1-Supplementary_Materials/Supplementary_Figures/Supplementary Figure S2.tif]

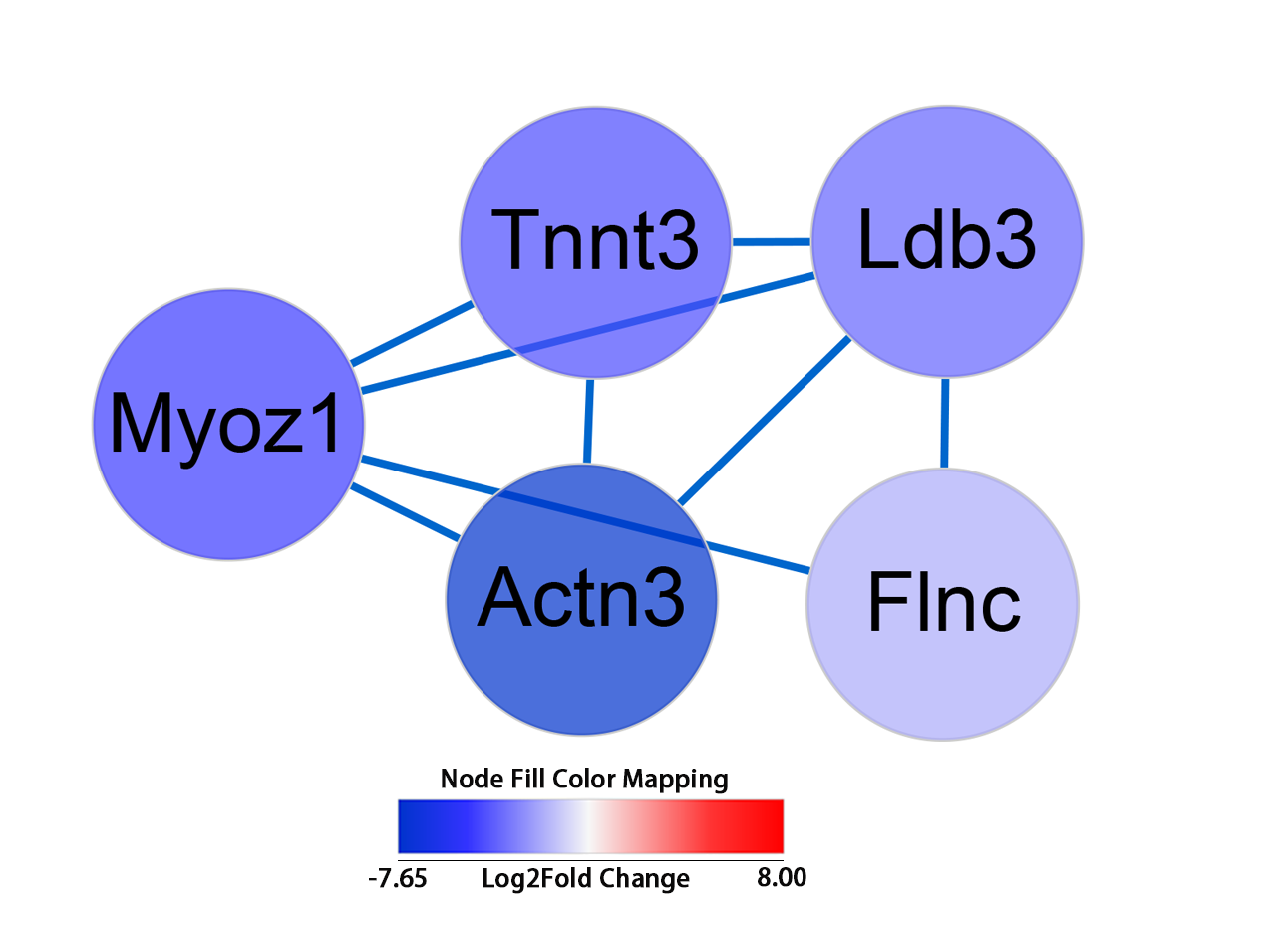

Supplement: Supplementary file 1 [file DataSheet1.zip › Data sheet 1-Supplementary_Materials/Supplementary_Figures/Supplementary Figure S3.tif]
